# PUMA-mediated apoptosis in fibroblast-like synoviocytes does not require p53

**DOI:** 10.1186/ar2052

**Published:** 2006-10-02

**Authors:** Xin You, David L Boyle, Deepa Hammaker, Gary S Firestein

**Affiliations:** 1 Division of Rheumatology, Allergy and Immunology, University of California at San Diego School of Medicine, 9500 Gilman Drive, La Jolla, California 92093, USA

## Abstract

*PUMA *(p53-upregulated modulator of apoptosis) is a pro-apoptotic gene that can induce rapid cell death through a p53-dependent mechanism. However, the efficacy of *PUMA *gene therapy to induce synovial apoptosis in rheumatoid arthritis might have limited efficacy if p53 expression or function is deficient. To evaluate this issue, studies were performed to determine whether p53 is required for PUMA-mediated apoptosis in fibroblast-like synoviocytes (FLS). p53 protein was depleted or inhibited in human FLS by using p53 siRNA or a dominant-negative p53 protein. Wild-type and p53^-/- ^murine FLS were also examined to evaluate whether p53 is required. p53-deficient or control FLS were transfected with PUMA cDNA or empty vector. p53 and p21 expression were then determined by Western blot analysis. Apoptosis was assayed by ELISA to measure histone release and caspase-3 activation, or by trypan blue dye exclusion to measure cell viability. Initial studies showed that p53 siRNA decreased p53 expression by more than 98% in human FLS. Loss of p53 increased the growth rate of cells and suppressed p21 expression. However, PUMA still induced apoptosis in control and p53-deficient FLS after PUMA cDNA transfection. Similar results were observed in p53^-/- ^murine FLS or in human FLS transfected with a dominant-negative mutant *p53 *gene. These data suggest that PUMA-induced apoptosis in FLS does not require p53. Therefore, approaches to gene therapy that involve increasing PUMA expression could be an effective inducer of synoviocyte cell death in rheumatoid arthritis regardless of the p53 status in the synovium.

## Introduction

Rheumatoid arthritis (RA) is a chronic inflammatory disease characterized by synovial hyperplasia and invasion into cartilage and bone. Inadequate apoptosis of fibroblast-like synoviocytes (FLS) could contribute to this process by increasing the accumulation of cells in the intimal lining [1]. As a result of the aggressive nature of rheumatoid synovium and the relatively low level of apoptosis, interventions designed to increase programmed cell death of synoviocytes have been considered in treating RA. Several genes have been evaluated as potential gene therapy targets, including *Fas *[2], *TRAIL *(tumor necrosis factor-related apoptosis-inducing ligand) [3], *p53 *[4], and *PUMA *(p53 up-regulated modulator of apoptosis) [5]. The latter is an especially interesting target because it rapidly induces apoptosis in cultured synoviocytes [5]. PUMA is a Bcl-2 homology 3 (BH3)-only pro-apoptotic Bcl-2 family member recently identified as a principal mediator of p53-dependent apoptosis [6]. The *in vivo *effects on apoptosis observed in PUMA^-/- ^mice are similar to those in p53^-/- ^animals, suggesting that PUMA can serve as an effector of p53 function [7,8]. However, our previous studies showed that p53 is only a weak inducer of PUMA in FLS, which could account for the variable pro-apoptotic effect of p53 in this cell lineage, with no significant apoptosis induced by p53 overexpression in some studies [9,10].

The mechanism of PUMA-mediated apoptosis has been extensively evaluated. PUMA expression leads to apoptosis by displacing p53 from Bcl-XL and allowing p53 to increase mitochondrial permeability [6]. The need for functional p53 raises significant concerns about the utility of PUMA as a therapeutic target in RA because deficient p53 expression or function in the rheumatoid synovial intimal lining has been described [11-14]. To address this issue, we determined whether PUMA requires functional p53 in cultured FLS. These studies show that PUMA-induced apoptosis can occur despite defects in the p53 pathway.

## Materials and methods

### Human and murine cultured fibroblast-like synoviocytes

Synovial tissues were obtained from patients with rheumatoid arthritis and osteoarthritis at joint replacement surgery. The diagnosis of RA conformed to the American College of Rheumatology 1987 revised criteria [15]. The protocol was approved by the University of California at San Diego Human Subjects Research Protection Program. FLS were isolated from individual tissues with 1 mg/ml collagenase and cultured in DMEM supplemented with 10% fetal calf serum, penicillin, streptomycin, and L-glutamine as described previously. Cell lines were used from the third to ninth passage, when they are a homogeneous population of fibroblast-like cells [16]. Although the origin of these cells cannot be certain, they probably derive from the intimal lining, on the basis of vascular cell adhesion molecule (VCAM)-1 and CD55 expression. In addition to RA FLS, we also examined FLS derived from osteoarthritis FLS in most experiments. No differences were observed between RA and osteoarthritis FLS in these assays. p53^+/+ ^and p53^-/- ^murine synoviocytes were obtained as described previously from DBA/1J wild-type mice (Jackson Laboratory, Bar Harbor, ME, USA) and DBA/1J p53^-/- ^mice [17].

### Antibodies

Affinity-purified rabbit polyclonal anti-p53 (for immunohistochemistry), mouse monoclonal anti-p53 (for Western blotting), and rabbit polyclonal antibodies against p21 and hemagglutinin (HA) were purchased from Santa Cruz Biotechnology (Santa Cruz, CA, USA). Anti-mouse and anti-rabbit IgG secondary antibodies were purchased from Cell Signaling Technology, Inc. (Beverly, MA, USA). Rabbit anti-PUMA polyclonal antibody was purchased from ProSci, Inc. (Poway, CA, USA).

### Cell transfections

Scrambled RNA and p53 siRNA were purchased from Dharmacon Research, Inc. (Lafayette, CO, USA). Plasmids encoding HA-tagged full-length PUMA (HA-PUMA) and PUMA with a deletion of the BH3 domain (HA-PUMA-dBH3) were kindly provided by Dr B Vogelstein (Johns Hopkins Oncology Center, Baltimore, MD, USA) [5]. R213* encoding mutant p53 was isolated from a patient with RA and has previously been characterized as dominant-negative [14]. Bax-luc (BF72-2 PGL3) is a reporter construct containing the p53-responsive promoter for *bax *with the luciferase cDNA [14]. The control construct contains the β-Gal cDNA and the cytomegalovirus (CMV) promoter in pCI. Cells were transfected with the use of the Amaxa Human Dermal Fibroblast Nucleofactor kit (NHDF-adult) with program U-23 for human FLS. Murine FLS were transfected with the use of the Mouse Embryonic Fibroblasts kit (MEF1) with program T-20. Cells (2 × 10^5 ^to 10^6^) were transfected with siRNAs, cDNAs, or control plasmids in each reaction.

### Western blot analysis

Cultured FLS were washed with phosphate-buffered saline, and protein was extracted with lysis buffer (50 mM HEPES pH 8.0, 150 mM NaCl, 1% Triton X-100, 10% glycerol, 1 mM MgCl_2_, 1.5 mM EDTA, 20 mM β-glycerophosphate, 50 mM NaF, 1 mM Na_3_VO_4_, 10 μg/ml aprotonin, 1 μM pepstatin A, 1 mM phenylmethylsulphonyl fluoride). The protein concentrations were determined with the DC protein assay kit (Bio-Rad, Hercules, CA, USA). Whole cell lysates containing 50 μg of protein were fractionated by 12% SDS-PAGE and transferred to a nitrocellulose membrane. The membrane was blocked with Tris-buffered saline plus 0.1% Tween 20 (TBST) containing 5% non-fat milk for 1 hour at room temperature followed by incubation overnight with the appropriate antibody at 4°C. The membrane was washed three times and incubated with horseradish peroxidase-conjugated secondary antibody for 1 hour. Immunoreactive protein was detected by chemiluminescence with Kodak X-AR film (Eastman Kodak, Rochester, NY, USA).

### Immunohistochemistry

siRNA-transfected cells for immunostaining were cultured in four-well chamber slides at 4.0 × 10^4 ^cells per well. They were then fixed with methanol, permeabilized with 0.05% Triton X-100 and blocked with 10% human serum. The fixed cells were incubated overnight with anti-p53 antibody or matched control antibody at 4°C. Endogenous peroxidase was then depleted with 0.1% H_2_O_2 _and 0.1% NaN_3_. The cells were then washed and stained with biotinylated secondary antibody anti-mouse or anti-rabbit IgG and Vectastain ABC and developed with diaminobenzidine (Vector, Burlingame, CA, USA).

### Cell viability and apoptosis assays

FLS were harvested and suspended in 0.2% trypan blue and counted with a hemocytometer. Cells that excluded dye were considered viable. Apoptosis was determined with a Cell Death Detection ELISA^PLUS ^kit (Roche Applied Science, Mannheim, Germany). FLS (4 × 10^3^) were seeded into each well of a 96-well plate after transfection. Nine hours later, samples were collected and ELISA was performed in accordance with the manufacturer's instructions. Results are presented as the fold induction compared with control. To confirm the role of apoptosis, caspase-3 activation was also determined in transfected cells with the use of the human active caspase-3 ELISA (R&D Systems, Minneapolis, MN, USA). PUMA or PUMA-dBH3 plasmids were transduced into p53 siRNA-transfected or scrambled siRNA-transfected cells. The cells were then cultured at 4.5 × 10^5 ^cells per well in six-well plates. Eight hours later, the cells were lysed and assayed as described by the manufacturer.

### Cell proliferation assay

Alamar Blue assays incorporate a fluorimetric/colorimetric growth indicator based on the detection of metabolic activity. FLS (3 × 10^3^) were plated into 96-well plate after siRNA transfection. At various time points, medium was replaced by DMEM without phenol red supplemented with 10% Alamar Blue. After incubation for 4 hours at 37°C, fluorescence was measured with a microplate reader at an excitation wavelength of 530 nm and an emission wavelength of 590 nm. The number of cells is expressed as relative fluorescence units.

### Statistical analysis

Data are expressed as means ± SEM. Statistics were performed with Student's *t *test, one-way analysis of variance and repeated-measures analysis of variance. A comparison was considered significant at *p *< 0.05.

## Results

### Use of siRNA to knockdown p53 protein in fibroblast-like synoviocytes

**Figure 1:**
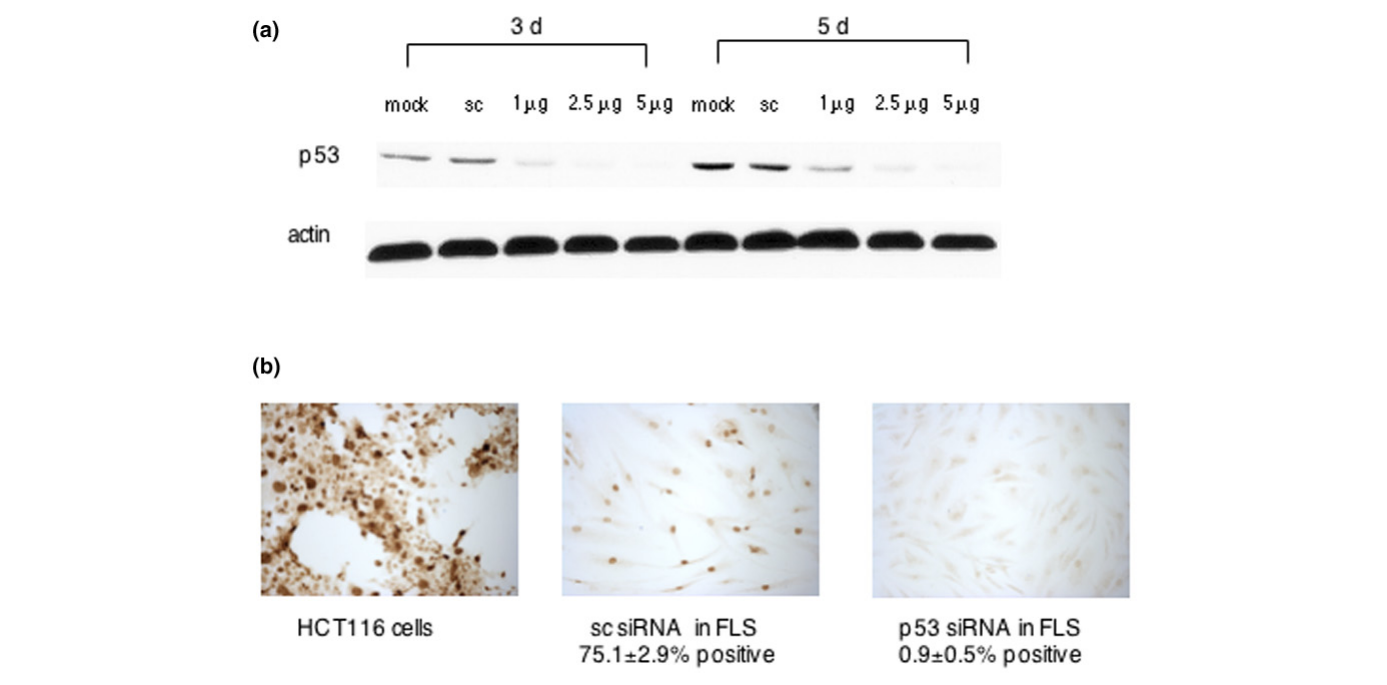
p53 knockdown by siRNA. **(a) **Western blot analysis. Cultured fibroblast-like synoviocytes (FLS) were transfected with 1, 2.5, or 5 μg of siRNA or non-silence scrambled siRNA (sc) as described in the Materials and methods section. Mock-transfected cells were treated in the same manner except that no siRNA was added. FLS were then incubated for 3 or 5 days and Western blot analysis was performed. The p53 level was decreased on day 3 and day 5, especially with the higher amounts of siRNA. For subsequent experiments, 2.5 μg of siRNA for 3 days was chosen as an optimum condition. Two FLS lines were studied with similar results. **(b) **Immunohistochemistry staining of p53 protein expression in rheumatoid arthritis FLS. Transfected FLS were seeded into four-well chamber slides, cultured for 5 days, and evaluated by immunohistochemistry. Prominent staining was observed in the positive control (colon tumor cell line HCT116) as well as scrambled siRNA-transfected FLS. The number of p53-positive cells was significantly reduced after transfection with 1 μg (not shown) or 5 μg of p53 siRNA (*p *< 0.0001 for p53 siRNA-transfected cells compared with scrambled siRNA). Percentages of p53-positive cells are shown as means ± SEM. Two FLS lines were studied with similar results. An irrelevant control antibody was positive in less than 1% of cells (not shown).

Initial studies were performed to determine whether siRNA could knock down p53 expression in cultured FLS. A representative time course and dose response are shown in Figure 1a. The residual expression of p53 protein was about 8 to 10%, 5%, and 1 to 2% for 1, 2.5, and 5 μg of siRNA, respectively, as determined by Western blot analysis. Immunohistochemistry also demonstrated a marked decrease in the percentage of p53-positive cells after siRNA transduction (Figure 1b). The percentage of cells with detectable p53 protein was 75.1 ± 2.9% for scrambled siRNA, 2.8 ± 0.6% for 1 μg of p53 siRNA, and 0.9 ± 0.5% for 5 μg of p53 siRNA.

### Functional effects of p53 deficiency

**Figure 2:**
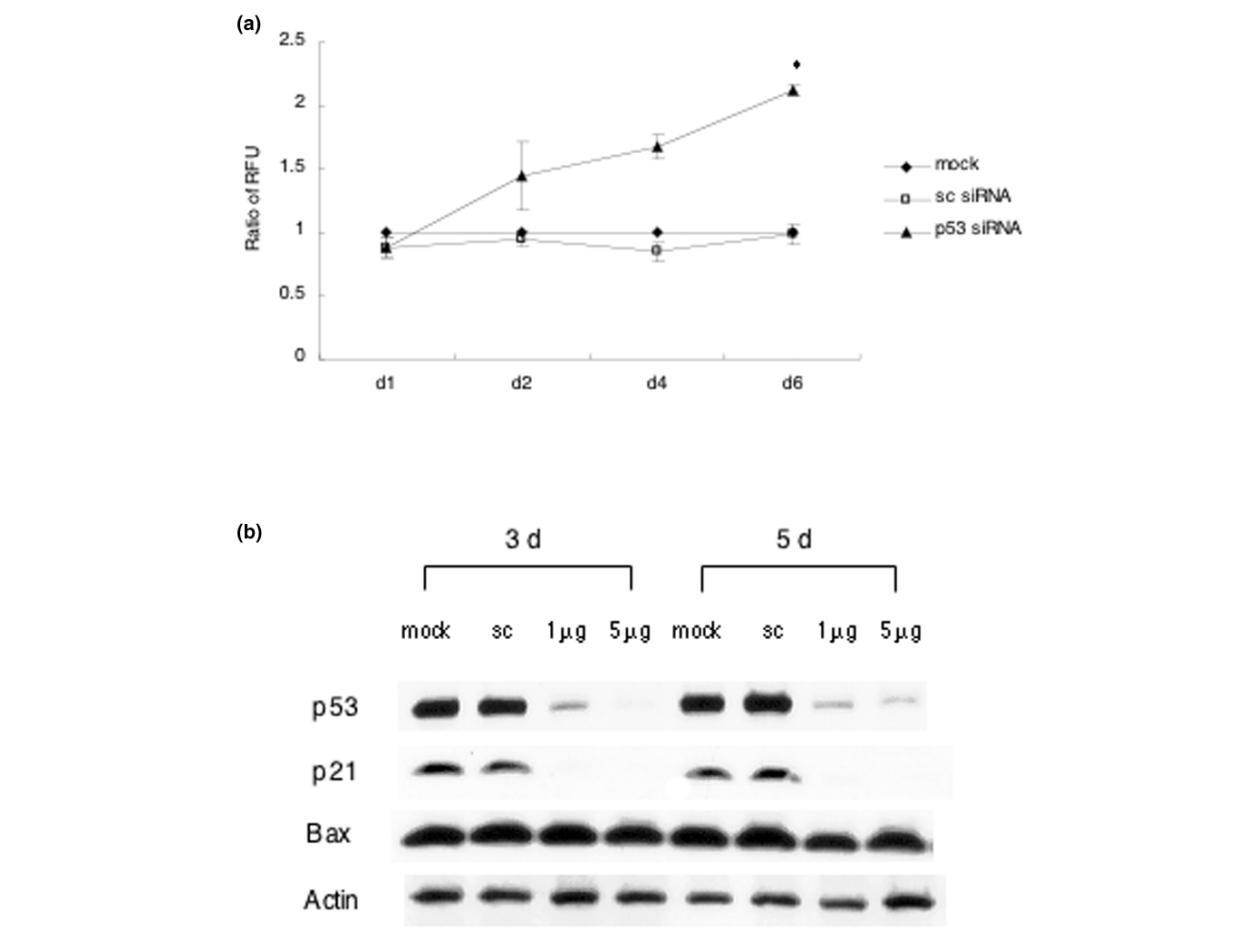
Effect of p53 siRNA on function of fibroblast-like synoviocytes (FLS). **(a) **Cell growth. p53 was knocked down by using siRNA, and cell growth was determined with an Alamar Blue assay in triplicate wells (*n *= 3 separate cell lines) The relative number of cells is quantified using arbitrary fluorescence units, and the fold induction in p53 siRNA-transfected FLS is shown relative to mock-transfected cells. Similar results were observed with 5 μg of siRNA (data not shown). *p < 0.001 for p53 siRNA-transfected cells compared with scrambled (sc) siRNA. RFU, relative fluorescence units. **(b) **p21 protein expression. p53 siRNA was knocked down by using siRNA, and p21 expression was determined by Western blot analysis. p21 expression decreased in cells with deficient p53 expression (*n *= 2 separate cell lines).

To confirm that p53 knockdown with siRNA was functionally relevant, the effect on cell proliferation and p21 expression was examined [18]. As shown in Figure 2a, growth of FLS was increased by p53 siRNA. Figure 2b shows that siRNA also blocked the expression of p21, which is normally induced by p53. These data indicate that p53 knockdown leads to functional alterations consistent with p53 deficiency.

### p53-independent apoptosis induced by PUMA

**Figure 3:**
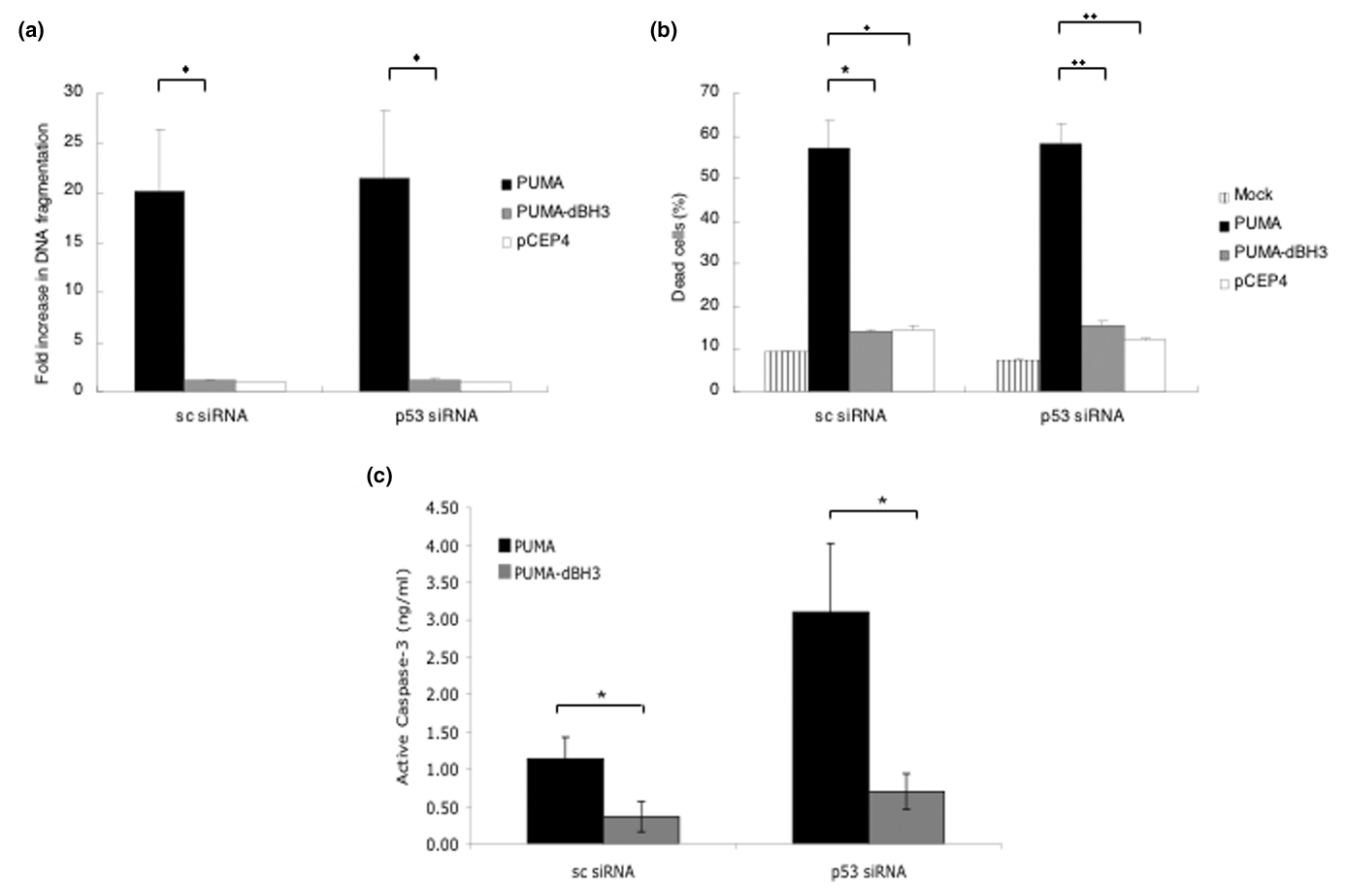
Apoptosis induced by PUMA in p53-deficient human fibroblast-like synoviocytes (FLS). Cultured FLS were transfected with siRNA and then, 3 days later, with 10 μg of PUMA, PUMA-dBH3, or pCEP4. **(a) **DNA fragmentation as determined by histone release. Histone release was measured by ELISA in samples collected 9 hours after the second transfection. The fold induction of DNA fragmentation in FLS transfected with PUMA plasmids is shown relative to the control value of pCEP4-transfected cells. *p < 0.05, *n *= 3. **(b) **Cell viability. Trypan blue exclusion was evaluated at 24 hours after cDNA transfection in 2.5 μg of siRNA-treated FLS. Data are presented as the percentage of non-viable cells. *p < 0.01, **p < 0.001, *n *= 3. **(c) **Caspase-3 activation. Caspase-3 levels were determined in control (sc) and p53-deficient (p53) FLS 8 hours after transfection with PUMA. *p < 0.05, *n *= 3.

To determine whether p53 is required for PUMA-induced apoptosis, FLS were transduced with p53 siRNA. After p53 expression reached its minimum 3 days later, cells were transfected with the PUMA cDNA, PUMA-dBH3, or empty plasmid pCEP4. As shown in Figure 3a,b, PUMA-induced apoptosis in scrambled siRNA-transfected cells, as measured by either histone release or cell viability, was similar to that in p53 siRNA-transfected cells. PUMA-dBH3, which lacks the BH3 domain and does not induce apoptosis, had no effect on cell viability. As further evidence of PUMA-mediated apoptosis, caspase-3 activation was also evaluated in p53-deficient FLS. Figure 3c shows that PUMA does not require p53 to engage this pathway.

### p53-independent apoptosis induced by PUMA in murine fibroblast-like synoviocytes

**Figure 4:**
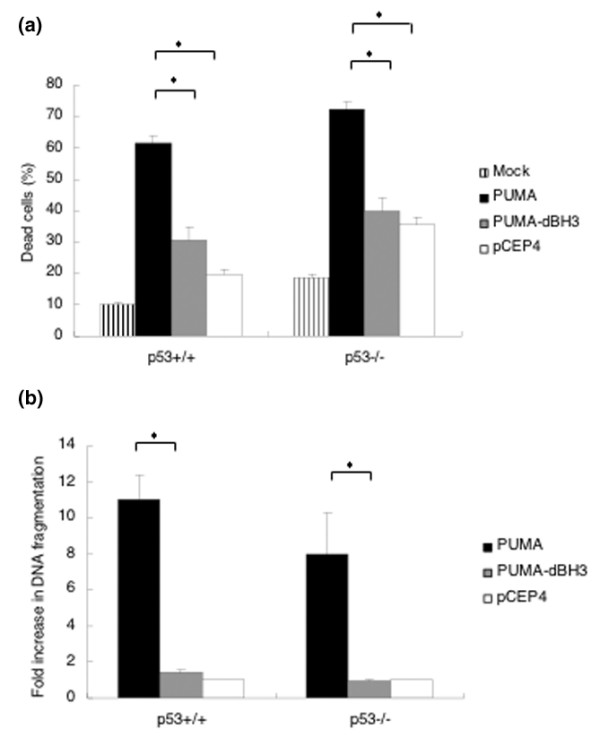
Apoptosis induced by PUMA in p53^+/+ ^and p53^-/- ^murine fibroblast-like synoviocytes (FLS). Murine p53^+/+ ^and p53^-/- ^FLS at passage 6 were transfected as described in the Materials and methods section. Cell viability by trypan blue staining **(a) **(*p < 0.05, *n *= 3) and histone release **(b) **(*p < 0.05, *n *= 3) was used to evaluate PUMA-induced apoptosis in both wild-type and p53-knockout FLS.

Small amounts of residual p53 might contribute to PUMA apoptosis in the siRNA studies, so we repeated the experiments in p53^+/+ ^and p53^-/- ^murine synoviocytes. Figure 4a shows that cell viability overall was decreased in the transfected cells. However, the effect of PUMA compared with PUMA-dBH3 or empty vector was the same in p53^+/+ ^and p53^-/- ^synoviocytes (Figure 4).

### PUMA-mediated apoptosis in the presence of mutant p53

**Figure 5:**
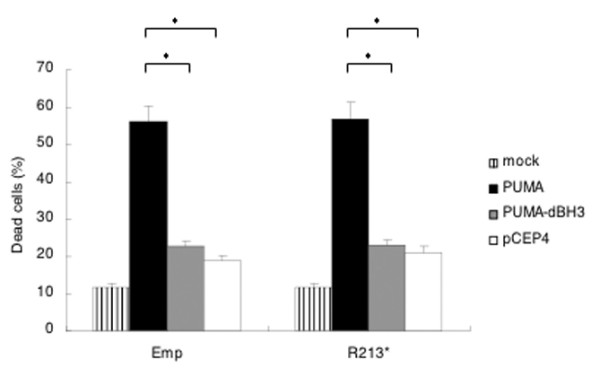
Apoptosis induced by PUMA in the presence of mutant p53. Human fibroblast-like synoviocytes (FLS) were transfected with dominant-negative p53 cDNA R213* or empty vector (Emp) followed by transfection with PUMA cDNA. Apoptosis was induced in FLS despite expression of the dominant-negative protein. *p < 0.05, *n *= 3.

Because some RA synoviocytes could potentially express dominant-negative p53 protein, we determined whether PUMA could induce cell death in the presence of mutant p53. A known dominant-negative gene that was isolated from a patient with RA (R213*) was used for these experiments [14]. Sequential transfection of cultured human FLS with R213* and followed with PUMA 2 days later was performed. Figure 5 shows that PUMA effectively induced apoptosis in FLS even in the presence of a dominant-negative p53.

## Discussion

Several therapeutic approaches to RA have focused on inducing apoptosis in the synovium, especially the intimal lining [2,4]. This region is populated by macrophage-like and fibroblast-like synoviocytes and is a primary source of cytokines and enzymes that degrade the extracellular matrix. The accumulation of cells in the lining can be due to ingress of cells from the blood, local proliferation, or insufficient deletion through apoptosis. The latter is especially intriguing in view of the observation that many pro-apoptotic genes are either defective or minimally expressed in RA, including *p53*, *sentrin *[19], and *PTEN *(phosphatase and tensin homologue deleted on chromosome 10 [20].

*p53 *is an interesting potential therapeutic gene because it can induce apoptosis in many cell types. Although controversial, defects in p53 structure and function in RA have been described, suggesting that forced expression of the tumor suppressor protein could be beneficial [11,12,21,22]. However, enhancing *p53 *gene expression in synovium with an adenoviral construct had only modest efficacy in a rabbit model of arthritis [4], and a similar approach was not effective in the rat adjuvant arthritis model (P.P. Tak, D.L. Boyle, G.S. Firestein, unpublished data). One potential explanation for the limited effect is that p53 does not readily induce apoptosis in synoviocytes, probably because PUMA expression is not increased [5]. In contrast, directly transducing cells with PUMA leads to rapid synoviocyte death *in vitro*.

One issue that could potentially interfere with the efficacy of *PUMA *gene therapy in RA is that this protein usually requires the p53 to induce apoptosis [6]. Elegant studies have demonstrated that the mechanism of PUMA action is through the release of p53 from inhibitory interactions with Bcl-XL in the cytoplasm [6]. Unbound p53 protein can then directly activate Bax. If p53 is defective or deficient, the benefit of forced PUMA expression would potentially be lost.

PUMA accounts for many of the apoptotic activities attributed to p53 [9,10], although it can serve as a mediator of some apoptotic pathways that do are not initiated by p53 induction, including glucocorticoids and serum deprivation [7,23]. PUMA-mediated apoptosis can also bypass p53 in unusual situations, especially in tumor cells. For instance, p53 expression does not require PUMA in melanoma and glioma cell lines [24,25] or human leukemia cells [26]. Hence, the utility of PUMA as an apoptosis-inducing protein and its relationship to p53 depends on the cell lineage, the status of p53 (deficiency versus mutation), and the type of stimulus. Therefore p53 has a dual role related to *PUMA *gene expression and function. In most cell types, p53 expression leads to increased *PUMA *gene expression and subsequent PUMA-mediated apoptosis requires functional p53. It is of interest that neither of these relationships is effective in cultured FLS. This cell lineage can also be distinguished from other cells in that p53 is expressed constitutively [18] even though the short half-life of wild-type p53 protein generally limits detection in non-cycling cells.

These highly variable data imply that tissue-specific cells should be studied to determine the potential applicability of *PUMA *gene therapy to RA. Our experiments using siRNA to decrease p53 expression show that FLS are very sensitive to PUMA-induced death and that p53 expression has no influence on this effect. Because siRNA does not completely deplete p53 levels, we confirmed these results in p53^-/- ^murine FLS. Finally, we showed that PUMA could function even in cells transfected with a known dominant-negative p53 mutant. These data demonstrate that PUMA-induced apoptosis in synoviocytes does not require p53 and that PUMA gene transfer could be effective regardless of the p53 status of the synovium.

These data support the potential use of *PUMA *as a local gene therapy approach to RA. By circumventing possible abnormalities in p53 and inducing extensive apoptosis of synoviocytes, intra-articular gene transfer could decrease the hyperplasia of the synovial intimal lining. Although not feasible for systemic administration, local therapy could debulk the synovium in RA and serve as an alternative to synovectomy or intra-articular corticosteroids.

## Conclusion

PUMA efficiently induced apoptosis in control and p53 deficient human FLS after PUMA overexpression. Similar results were observed in p53^-/- ^murine FLS and in human FLS transfected with the R123* mutant *p53 *gene. PUMA-induced apoptosis is therefore independent of p53 in FLS. These data suggest that *PUMA *gene therapy could be effective in RA regardless of the p53 status of the synovium.

## Abbreviations

BH3 = Bcl-2 homology 3; DMEM = Dulbecco's modified Eagle's medium; ELISA = enzyme-linked immunosorbent assay; FLS = fibroblast-like synoviocytes; HA = hemagglutinin; PUMA = p53-upregulated modulator of apoptosis; RA = rheumatoid arthritis; siRNA = small interfering RNA.

## Competing interests

The authors declare that they have no competing interests.

## Authors' contributions

XY performed experiments, evaluated data and wrote the manuscript. DLB designed experiments and evaluated data. DH performed experiments and evaluated data. GSF designed experiments, evaluated data, and wrote the manuscript. All authors read and approved the final manuscript.
